# Concurrent healthy behavior adoption and diabetic retinopathy in the United States

**DOI:** 10.1016/j.pmedr.2015.07.002

**Published:** 2015-07-18

**Authors:** Paul D. Loprinzi

**Affiliations:** Center for Health Behavior Research, Department of Health, Exercise Science, and Recreation Management, The University of Mississippi, 229 Turner Center, University, MS 38677, United States

**Keywords:** Epidemiology, Diabetes, Diet, Physical activity, Retinopathy

## Abstract

**Objective:**

Emerging work suggests an independent association of physical activity and healthy eating on diabetic retinopathy. No study, however, has examined whether physical activity and healthy eating have an additive and/or additive interaction effect on diabetic retinopathy.

**Methods:**

Data from 2005–2006 NHANES were used (N = 223). Physical activity was assessed via accelerometry; healthy eating was assessed from an interview with the Healthy Eating Index calculated to represent healthy eating; and diabetic retinopathy was assessed from the Canon Non-Mydratic Retinal Camera CR6-45NM.

**Results:**

Physical activity (OR = 0.70, p = 0.42) and healthy eating (OR = 0.36, p = 0.16) were not independently associated with moderate-to-severe retinopathy. However, individuals with both health behaviors, compared to none, had a reduced odds of moderate-to-severe retinopathy (OR = 0.03, p = 0.02). Further, the attributable proportion (AR = 0.57, 95% CI 0.02–1.12, p < 0.05) was significant, suggesting that a significant proportion of retinopathy may be attributed to the additive interaction between inactivity and unhealthy eating.

**Conclusion:**

The concurrent presence of physical activity and healthy eating was associated with reduced odds of diabetic retinopathy, with the additive interaction effects suggesting that this observed association is more than summation.

## Highlights

• National epidemiological study was employed.
• Objective measures of physical activity and retinopathy were employed.
• Combined healthy eating and physical activity reduced odds of retinopathy.

## Introduction

Emerging research suggests that regular participation in physical activity may be associated with reduced odds of having diabetic retinopathy (Dirani et al., 2014, Loprinzi et al., 2014a), as well as other ocular-related parameters, such as visual impairment (Loprinzi et al., 2014b) and age-related macular degeneration (Loprinzi et al., 2015, Knudtson et al., 2006). Although the mechanisms are unknown at this point, potential reasons to explain this potential relationship include physical activity-induced modulation of parameters (e.g., glycemic control and blood pressure) known to increase the risk of developing diabetic retinopathy (Ding & Wong, 2012). Through similar potential mechanisms, emerging work is also demonstrating that the broad consumption of a healthy diet (e.g., greater adherence to dietary guidelines) is associated with reduced odds of diabetic retinopathy (Cundiff and Nigg, 2005, Mahoney and Loprinzi, 2014). (See Table 1, Table 2.)

Taken together, this emerging work suggests that both physical activity and healthy eating are associated with diabetic retinopathy. Although it is plausible to suggest that physical activity and diet would have an additive and/or additive interaction effect on diabetic retinopathy, no studies to date have examined this possibility, but rather, just examined their independent effects. As a result, the purpose of this study was to examine the potential additive and additive interaction effects of physical activity and healthy eating on the odds of diabetic retinopathy.

## Methods

### Study design and participant

Data were restricted to the 2005–2006 NHANES cycle because this is the only wave with objectively-measured physical activity (i.e., accelerometry) and retinopathy data. The NHANES is an ongoing survey conducted by the Centers for Disease Control and Prevention that uses a representative sample of non-institutionalized United States civilians selected by a complex, multistage, stratified, clustered probability design. In brief, participants were interviewed in their homes and then subsequently examined in a mobile examination center (MEC) by NHANES personnel. Further details about NHANES can be found elsewhere. NHANES study procedures were approved by the National Center for Health Statistics ethics review board, with informed consent obtained from all participants prior to data collection.

In the 2005–2006 NHANES, 521 participants had a physician diagnosis of diabetes, with 513 providing data on their diabetes duration. Only those ≥ 40 yrs were eligible for retinopathy assessment, which included 461 adults above or equal to this age. Among these 461 participants, 347 provided retinopathy data. After excluding participants with missing data on the covariates, 319 remained. After excluding those with missing dietary data, 312 remained. Lastly, after excluding those with insufficient accelerometry data (< 4 days or 10 + h/day of monitoring), 223 participants remained, which constituted the analytic sample.

Previous studies have shown that adults with invalid accelerometry data have different demographic, behavioral and health characteristics than their counterparts who provide valid accelerometry data (i.e., wear the monitor for at least 4 days of 10 + h/day) (Loprinzi et al., 2013). When comparing the 89 participants who were excluded due to missing or insufficient accelerometry data to the 223 analyzed participants, there were no differences in any of the study variables with the exception of age and body mass index (BMI); excluded participants were younger (59.9 yrs vs. 64.1 yrs, p = 0.001) and had a higher BMI (32.9 kg/m^2^ vs. 31.1 kg/m^2^, p = 0.04). These are unweighted estimates.

### Assessment of diabetic retinopathy

Participants aged 40 years and older were eligible for the retinal imaging exam unless they were unable to see light with both eyes open or had an eye infection. Detailed procedures of the retinal imaging exam performed in the NHANES 2005–2006 cycle can be found elsewhere (Zhang et al., 2010). Briefly, retinal imaging was performed using the Canon Non-Mydratic Retinal Camera CR6-45NM (Canon, Tokyo, Japan). Two forty-five degree non-mydriatic digital images were obtained on both eyes. Diabetic retinopathy was defined as the presence of 1 or more retinal microaneurysms or retinal blot hemorrhages using the Early Treatment Diabetic Retinopathy Study (ETDRS) grading criteria, and was further classified as *no retinopathy*, *mild non-proliferative retinopathy*, *moderate-to-severe non-proliferative retinopathy*, *or proliferative retinopathy* according to ETDRS standards applied to the worse eye. Notably, after exclusions, only 5 participants had proliferative retinopathy. Analyses were computed when these 5 participants were excluded and when they were collapsed into the moderate-to-severe non-proliferative group. Results were similar (data not shown); therefore, results are presented with these 5 participants included into the moderate-to-severe non-proliferative group.

### Measurement of physical activity

While attending the MEC, participants were instructed to wear an ActiGraph 7164 accelerometer during all activities, except water-based activities and while sleeping. The accelerometer measured the frequency, intensity, and duration of physical activity by generating an activity count proportional to the measured acceleration. Detailed information on the ActiGraph accelerometer can be found elsewhere (Chen & Bassett, 2005). Estimates for moderate-to-vigorous physical activity (MVPA) were summarized in 1-minute time intervals. Activity counts/min greater than or equal to 2020 were classified as MVPA (Troiano et al., 2008). For the analyses described here, and to represent habitual physical activity patterns, only those participants with activity patterns for at least 4 days of 10 or more hours per day of monitoring data were included in the analyses (Troiano et al., 2008). To determine the amount of time the monitor was worn, nonwear was defined by a period of a minimum of 60 consecutive minutes of zero activity counts, with the allowance of 1–2 min of activity counts between 0 and 100 (Troiano et al., 2008).

The SAS (version 9.2) was used to reduce the accelerometry data using the SAS code provided the National Cancer Institute. Using the SAS code, the average time each participant spent per day in physical activity was analyzed from valid individual data.

### Measurement of dietary behavior

Two 24-hour recall assessments of food and fluid intake were collected during the participant's visit to a MEC. To capture intake on all days of the week, the 24 hour recalls were collected on every day of the week. The dietary interviewers used the Dietary Data Collection (DDC) system, which is an automated standardized interactive dietary interview and coding system. The Healthy Eating Index (HEI) 2005 was developed by the USDA as an indicator of dietary quality (Guenther et al., 2007). The HEI is comprised of 12 components (total fruit; whole fruit; total vegetable; dark green, orange vegetable and legumes; total grain; whole grain; milk; meat and beans; oil; saturated fats; sodium; and calories from solid fats, alcoholic beverages, and added sugars) with each component individually scored, with a maximum total score of 100. A higher score reflects closer adherence to the dietary guidelines for Americans. The HEI was derived for each of the 24 hour recall days using the MyPyramid Equivalents Database and following the methods and SAS code established by the USDA Center for Nutrition Policy and Promotion (Bowman et al., 2008). An average HEI across the two days were calculated and used.

### Calculation of concurrent Healthy Behavior Index

Using the average of the two-day HEI scores, and consistent with other studies (Ford et al., 2012, Loprinzi et al., 2014c), participants at or above the 60th percentile (i.e. top 40%) of HEI scores in the sample were categorized as consuming a healthy diet. With regard to physical activity, few participants (n = 27) meet current physical activity guidelines (≥ 150 min/wk of MVPA); therefore, and similar to the calculation of ‘healthy eating’, participants were categorized as ≥ 60th percentile (i.e. top 40%) and < 60th percentile of MVPA.

Participants were classified as having 0–2 positive health behaviors by summing the number of health behaviors they had; for example those above the 60th percentile for *both* HEI and MVPA were considered to have 2 positive health behaviors.

### Measurement of covariates

Based on previous research demonstrating an association with physical activity, diet and retinopathy (Ding & Wong, 2012), covariates included age, sex, race-ethnicity, smoking, total cholesterol, HgbA1c, BMI, diabetes duration, visual acuity, and physician diagnosis of coronary heart disease, stroke, and hypertension.

Serum cotinine was measured by an isotope dilution-high performance liquid chromatography/atmospheric pressure chemical ionization tandem mass spectrometry. Total cholesterol was assessed enzymatically in serum or plasma. HgbA1C was measured using the Primus instrument, which is a fully automated glycohemoglobin analyzer using high performance liquid chromatography. BMI was calculated from measured weight and height (weight in kilograms divided by the square of height in meters). Details regarding the NHANES vision assessment are described elsewhere (Willis et al., 2012). For the present study, visual acuity was taken as the better-eye presenting acuity when no autorefraction was performed, and as the better-eye post-autorefraction visual acuity when autorefraction was performed. Visual acuity of the better-eye was then treated as a continuous variable expressed in logMAR units (logarithm of the minimum angle of resolution).

### Data analysis

Statistical analyses (Stata, version 12.0, College Station, TX) accounted for the complex survey design used in NHANES (analyzed in 2015). To account for oversampling, non-response, non-coverage, and to provide nationally representative estimates, all analyses included the use of survey sample weights, stratum and primary sampling units. Recalculated sample weights for the subsamples with 4 or more days of valid accelerometer data were used to make the selected samples nationally representative.

Multivariable polytomous regression analysis was used to examine the association between the number of positive health behaviors (independent variable) and diabetic retinopathy (outcome variable), with no retinopathy serving as the referent group. Statistical significance was set at p < 0.05.

In addition to examining a potential combined effect of physical activity and diet on diabetic retinopathy, further analyses were computed to examine any potential additive interaction effect of physical inactivity and unhealthy eating on any degree of retinopathy; the analytical approach as shown below requires a dichotomous outcome variable so, for these analyses, retinopathy was recoded as no retinopathy and mild or greater retinopathy. As described by Kalilani and Atashili (Kalilani & Atashili, 2006), and broadly speaking, additive interaction exists when the joint effect of the risk factors differs from the *sum* of the effects of the individual factors; the risk factors in this case were physical inactivity (< 60th percentile for MVPA) and unhealthy eating (< 60th percentile for HEI). Additive interaction was tested by calculating the relative excess risk due to interaction (RERI) and the attributable proportion due to interaction (AP) (Andersson et al., 2005). The RERI can be interpreted as the risk that is additional to the risk that is expected on the basis of the addition ORs under exposure, and AP is interpreted as the proportion of the condition that is due to interaction among persons with both exposures (de Mutsert et al., 2009).

RERI was calculated as: OR_++_ − OR_+−_ − OR_−+_ + 1; AP was calculated as RERI / OR_++_, where a category of joint exposure to both risk factors is denoted as (++) and a category of exposure to one of the risk factors only is denoted as (+− or −+).

In the absence of an additive interaction effect, RERI and AP equal 0. Therefore, evidence of significant additive interaction was determined when the 95% confidence intervals departed from 0 for AP and RERI (i.e., the lower bound CI was > 0).

## Results

Weighted characteristics of the study variables are shown in Table 1. Approximately 63%, 25%, and 12%, respectively, had no retinopathy, mild retinopathy, and moderate-to-severe retinopathy. Participants were, on average, 62 years old, and engaged in 11 min/day of MVPA.

In an adjusted polytomous model that had the binary (≥ and < 60th percentile) MVPA and healthy eating variables, MVPA (OR = 0.87, p = 0.81) and healthy eating (OR = 0.57, p = 0.45) were not independently associated with mild retinopathy compared to no retinopathy; similarly, MVPA (OR = 0.70, p = 0.42) and healthy eating (OR = 0.36, p = 0.16) were not independently associated with moderate-to-severe retinopathy (data not shown in tabular format).

Although there was no evidence of an independent association of MVPA and healthy eating, there was evidence of an additive effect in that individuals with both health behaviors had reduced odds of moderate-to-severe retinopathy. As shown in Table 2, and after adjustments, individuals with both health behaviors, compared to none, had a reduced odds of moderate-to-severe retinopathy (OR = 0.03, p = 0.02), but not mild retinopathy (OR = 0.61, p = 0.55).

To determine whether there was additive interaction (i.e., the joint effect is greater than the sum of the individual effects) between MVPA and diet on retinopathy, further analyses were performed. The RERI (RERI = 1.43, 95% CI: − 0.32–3.19) was not statistically significant, but did occur in the expected direction; the AP (AP = 0.57, 95% CI 0.02–1.12) was significant, suggesting that a significant proportion of retinopathy may be attributed to the additive interaction between inactivity and unhealthy eating. More specifically, both behaviors acted synergistically in relation to risk of retinopathy, so that the AP to retinopathy was 57% higher than expected from the addition of separate effects of physical inactivity and unhealthy eating. Put in another way, these two unhealthy behaviors (inactivity and unhealthy eating) interact to substantially increase the odds of diabetic retinopathy, *an effect that is more than summation*.

## Discussion

Previous research has demonstrated independent effects of physical activity and dietary behavior on diabetic retinopathy, as well as other ocular related parameters. However, to our knowledge, this is the first investigation of whether physical activity and dietary behavior have an additive and/or additive interaction effect on diabetic retinopathy. Our findings contribute to the literature by demonstrating that adults with diabetes who are relatively active and eat a healthy diet (i.e., concurrent healthy behavior adoption) have reduced odds of moderate-to-severe retinopathy and that concurrent healthy behavior adoption has an additive interaction effect on the reduced odds of retinopathy.

Presently, HgbA1c and blood pressure are the only reversible risk factors associated with diabetic retinopathy (Ding & Wong, 2012). Both physical activity and an overall healthy eating pattern are inversely associated with HgbA1c and blood pressure (Cornelissen and Smart, 2013, Appel et al., 2006, Delahanty and Halford, 1993, Sigal et al., 2006), which suggests a potential biological explanation for the health behavior-diabetic retinopathy relationship. However, our observed association persisted even after adjusting for predictors of prevalent retinopathy, including higher HbA1C and blood pressure. Physical activity and healthy eating also benefit vascular endothelial function (Loprinzi & Cardinal, 2012), and these benefits may be partially independent of blood pressure and HgbA1c, providing an explanation for our findings showing that concurrent behavioral presence was associated with diabetic retinopathy independent of HgbA1c and blood pressure.

Limitations of this study include the cross-sectional design, rendering temporal sequence not possible. As a result, future prospective work examining the association between the concurrent adoption of physical activity and healthy eating on diabetic retinopathy is warranted. Major strengths of this study include the study's novelty (examining additive and additive interaction effects), utilization of an objective measure of physical activity and diabetic retinopathy, and employing a national sample of adults with diabetes.

In conclusion, we did not observe independent associations of physical activity and healthy eating on diabetic retinopathy, but a combined/additive effect was observed in that those who were active and ate a healthy diet had a reduced odds of diabetic retinopathy. We also observed an additive interaction effect suggesting that this observed association is more than summation. If confirmed by future work, then studies examining potential explanations for the possible synergistic effects of physical activity and healthy eating on diabetic retinopathy are warranted.

## Conflict of interest

“The authors declare that there are no conflicts of interests.”

## Figures and Tables

**Table 1:** Weighted characteristics of the analyzed sample of U.S. diabetics, NHANES 2005–2006 (N = 223).

Variables	Mean/proportion (95% CI)
Non-proliferative diabetic retinopathy, %	
No retinopathy	63.3 (56.6–70.1)
Mild retinopathy	24.5 (20.6–28.4)
Moderate-to-severe retinopathy a	12.1 (7.1–17.1)
Health behaviors, mean	
Total MVPA, min/day	11.07 (8.08–14.06)
Mean MVPA for those ≥ 60th percentile	21.3 (17.1–25.5)
Mean MVPA for those < 60th percentile	2.8 (2.4–3.1)
HEI	57.4 (55.8–58.9)
Mean HEI for those ≥ 60th percentile	67.4 (65.6–69.2)
Mean HEI for those < 60th percentile	49.7 (48.3–51.1)
Concurrent healthy behavior index, mean	0.88 (0.75–1.00)
% 0 health behaviors	32.7 (27.6–37.9)
% 1 health behaviors	46.4 (40.1–52.6)
% 2 health behaviors	20.8 (12.6–28.9)
Age, yr	62.6 (60.5–64.6)
Race-ethnicity, %	
Mexican American	7.7 (3.5–12.0)
Non-Hispanic White	67.9 (58.2–77.7)
Non-Hispanic Black	16.2 (8.6–23.8)
Other	8.0 (1.7–14.2)
Gender, %	
Male	46.3 (37.5–55.1)
Female	53.6 (44.8–62.4)
Coronary heart disease, %	12.5 (7.4–17.7)
Stroke, %	10.4 (6.0–14.8)
Hypertension, %	65.1 (57.7–72.4)
Cotinine, ng/mL	37.2 (24.1–50.2)
Vision, logMAR	0.59 (0.39–0.78)
HgbA1c, %	6.95 (6.6–7.2)
Total cholesterol, mg/dL	188.4 (179.5–197.3)
BMI, kg/m^2^	32.3 (30.9–33.8)
Diabetes duration, yrs	10.0 (8.3–11.6)

MVPA = moderate-to-vigorous physical activity.

HEI = Healthy Eating Index.

a As noted in the methods section, the 5 participants who had proliferative retinopathy were recoded into the moderate-to-severe non-proliferative group.

**Table 2:** Polytomous model describing the association between the Health Behavior Index^b^ variable (independent variable) and the presence of either mild or moderate/severe diabetic retinopathy, compared to no retinopathy, NHANES 2005–2006 (N = 223).

Health Behavior Index b	Odds ratio (95% CI) a	
Mild NPDR	Moderate-to-severe NPDR
1 vs. 0	0.28 (0.07–1.13)	0.84 (0.17–4.07)
2 vs. 0	0.61 (0.10–3.40)	**0.03 (0.02–0.60)**
*Covariates*		
Age, 1 yr	1.01 (0.98–1.05)	0.96 (0.92–1.01)
Race-ethnicity		
Mexican American vs. Non- Hispanic White	1.12 (0.22–5.63)	0.59 (0.03–9.29)
Non-Hispanic Black vs. Non-Hispanic White	0.96 (0.28–3.28)	1.02 (0.21–4.89)
Other vs. Non-Hispanic White	1.62 (0.41–6.34)	0.49 (0.04–4.87)
Female vs. male	0.47 (0.18–1.21)	**0.29 (0.09–0.92)**
Coronary heart disease vs. none	1.50 (0.29–7.69)	1.09 (0.27–4.40)
Stroke vs. no stroke	1.11 (0.13–9.03)	0.88 (0.16–4.67)
Hypertension vs. none	1.77 (0.66–4.69)	1.25 (0.33–4.71)
Cotinine, 1 ng/mL	1.00 (0.99–1.00)	1.00 (0.99–1.01)
Vision, 0.1 logMAR units	0.96 (0.53–1.74)	**1.45 (1.01–2.08)**
HgbA1c, 1%	**1.73 (1.19–2.52)**	**2.26 (1.33–3.82)**
Total cholesterol, 1 mg/dL	0.99 (0.98–1.01)	0.99 (0.98–1.01)
BMI, 1 kg/m^2^	0.99 (0.92–1.06)	1.03 (0.96–1.12)
Diabetes duration, 1 yr	1.07 (0.98–1.17)	**1.08 (1.00–1.17)**

HgbA1c = hemoglobin A1C.

logMAR = logarithm of the minimum angle of resolution.

NPDR = non-proliferative diabetic retinopathy.

Bold = statistical significant association (p < 0.05).

a Not having diabetic retinopathy served as the referent group. All results are weighted.

b Participants were classified as having 0–2 positive health behaviors by summing the number of health behaviors they had, with having a positive health behavior being defined as at or above the 60th percentile for that behavior; for example those above the 60th percentile for both HEI (healthy eating index) and MVPA (moderate-to-vigorous physical activity) were considered to have 2 positive health behaviors.
